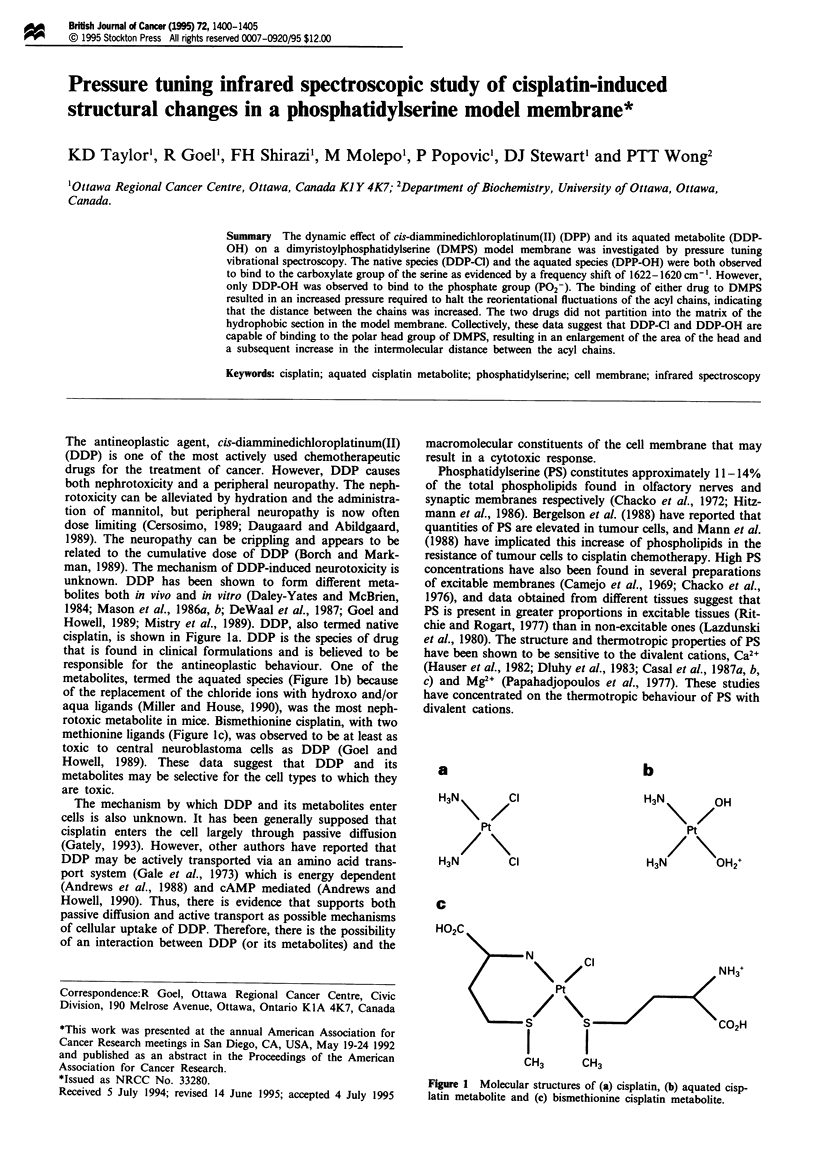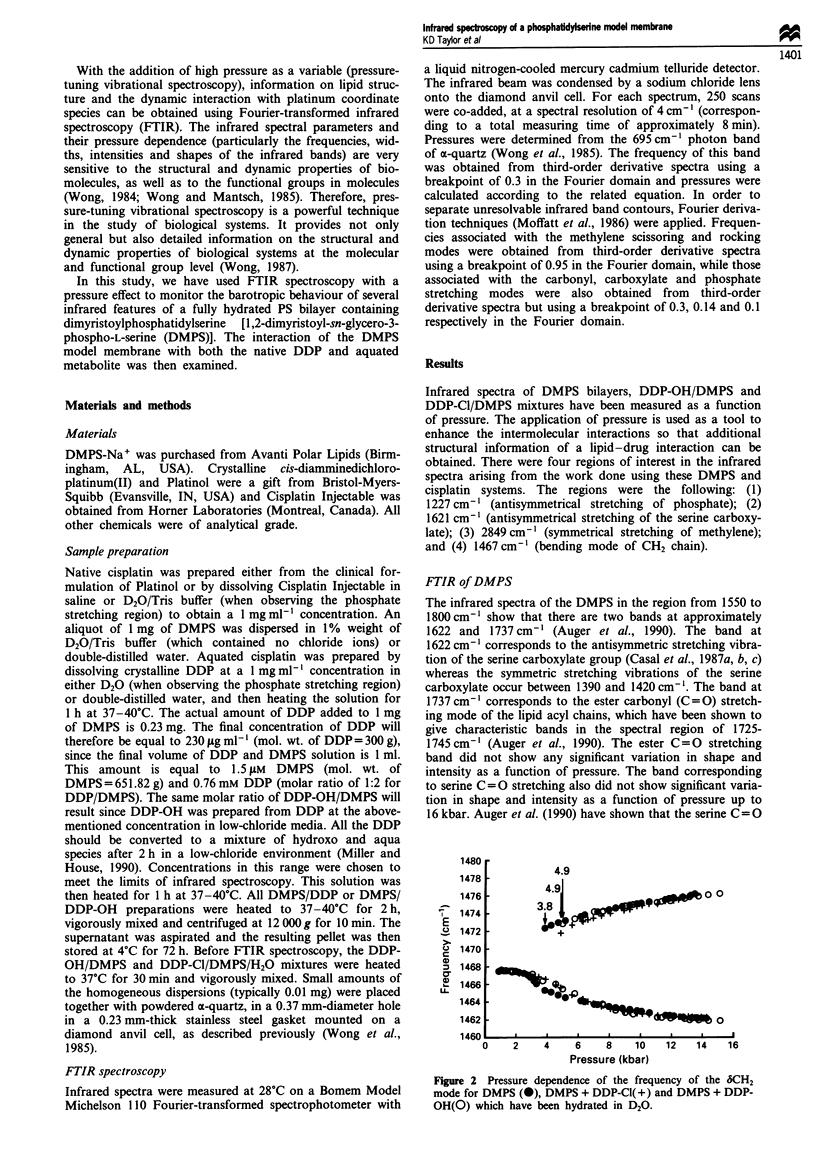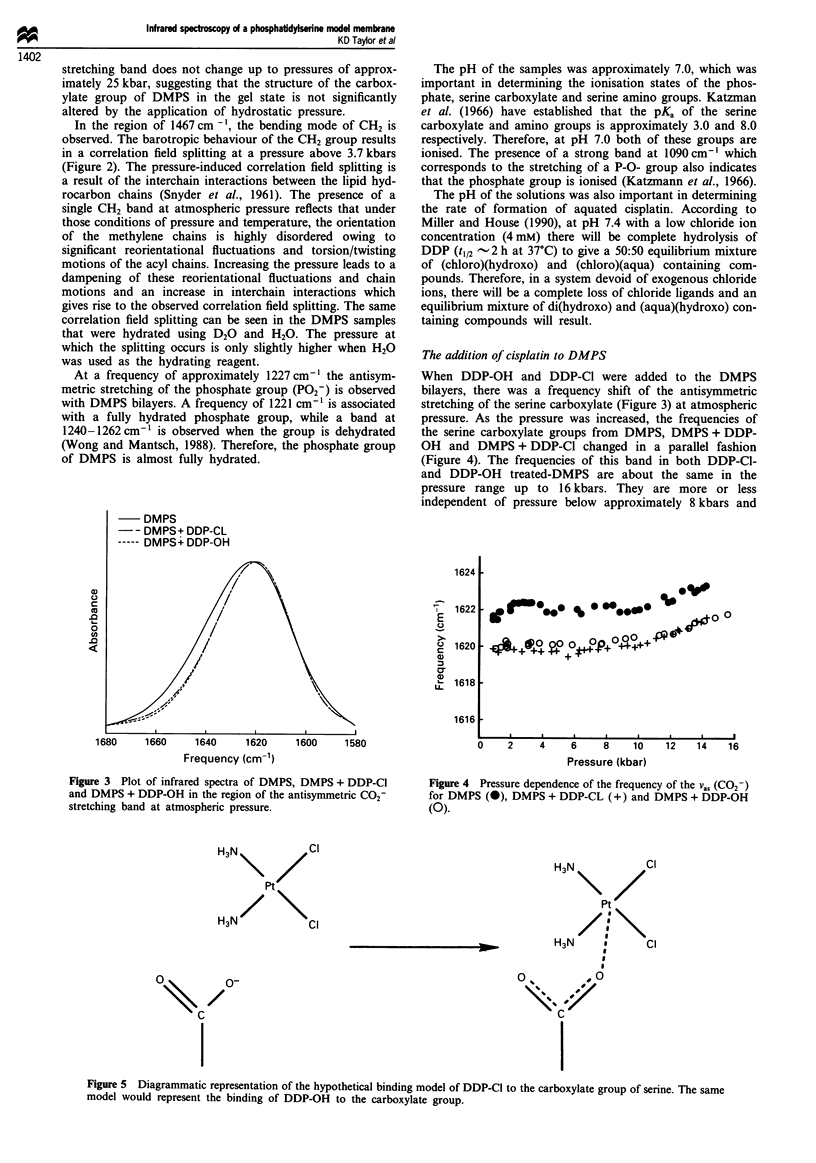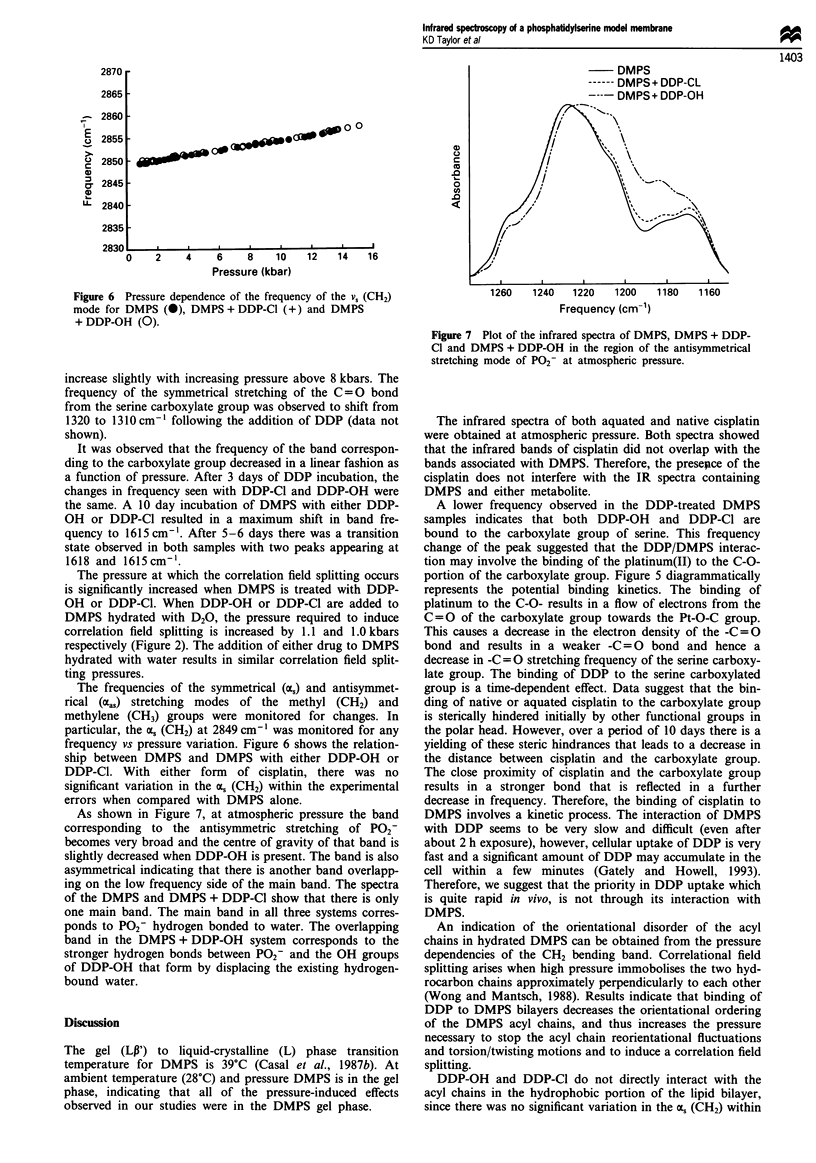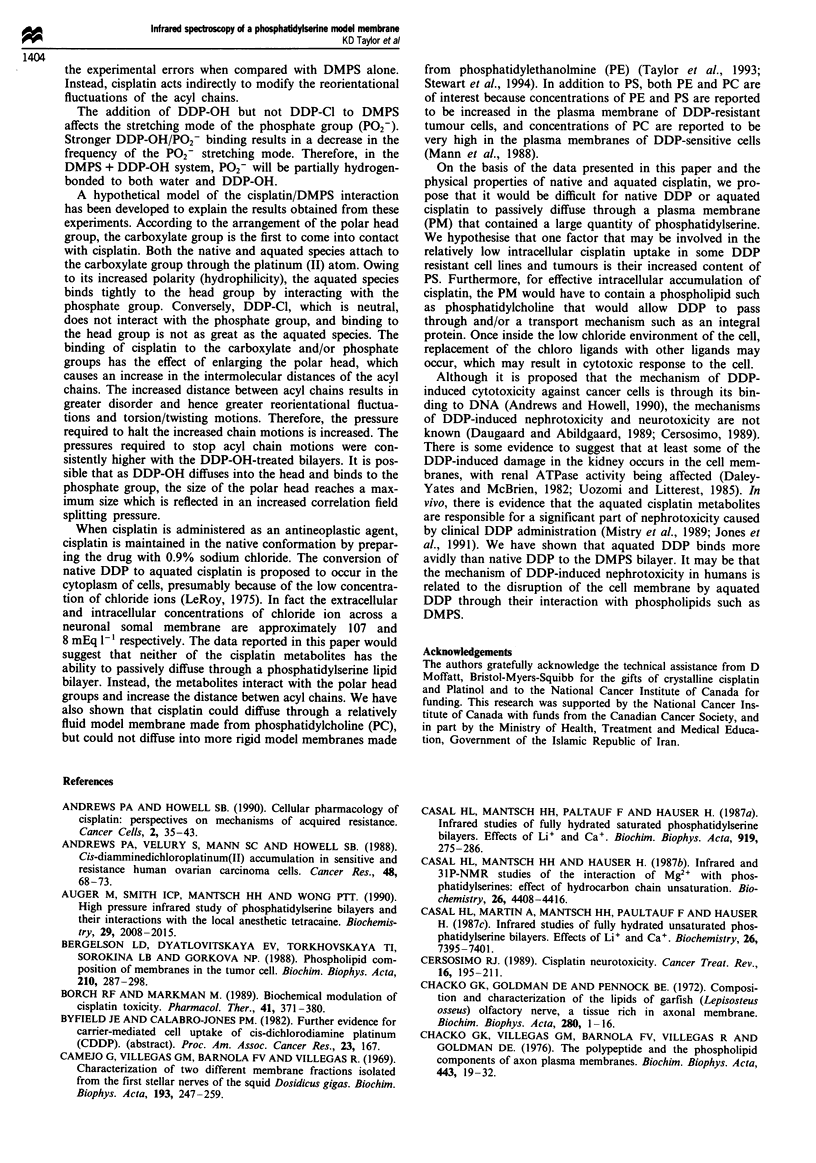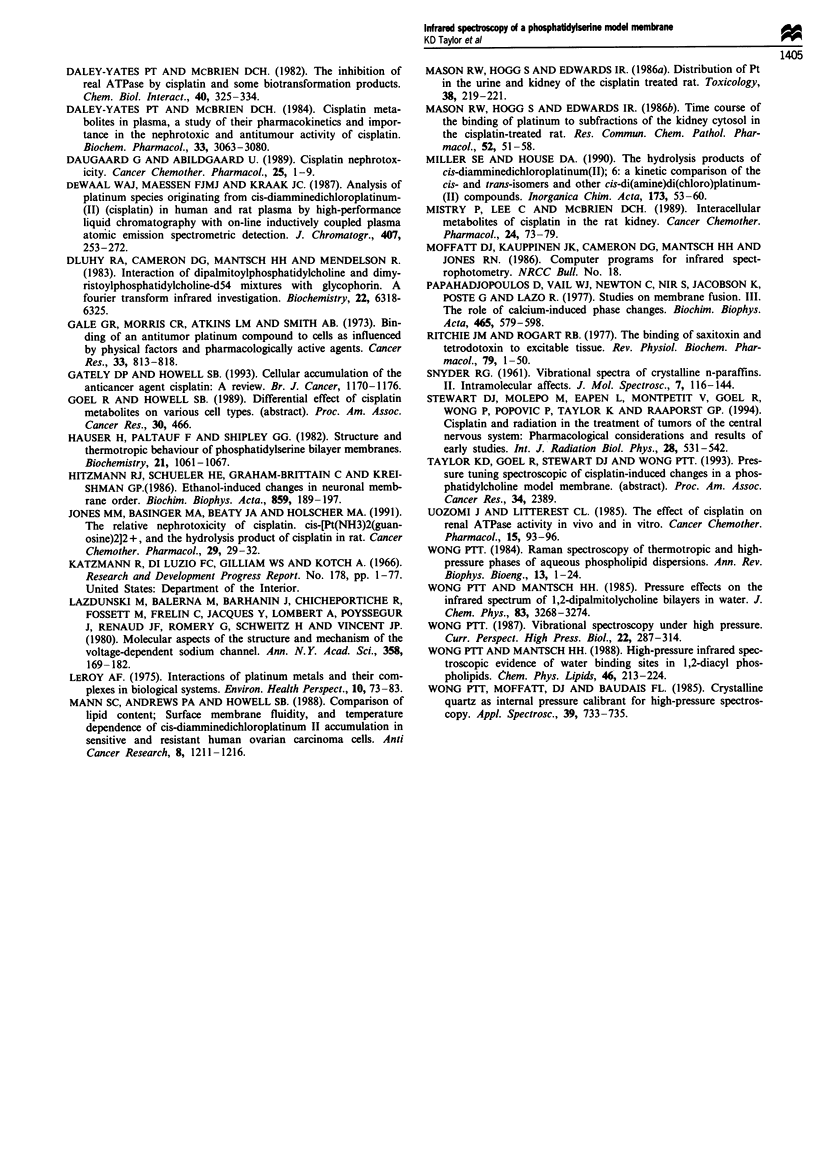# Pressure tuning infrared spectroscopic study of cisplatin-induced structural changes in a phosphatidylserine model membrane.

**DOI:** 10.1038/bjc.1995.521

**Published:** 1995-12

**Authors:** K. D. Taylor, R. Goel, F. H. Shirazi, M. Molepo, P. Popovic, D. J. Stewart, P. T. Wong

**Affiliations:** Ottawa Regional Cancer Centre, Canada.

## Abstract

The dynamic effect of cis-diamminedichloroplatinum(II) (DPP) and its aquated metabolite (DDP-OH) on a dimyristoylphosphatidylserine (DMPS) model membrane was investigated by pressure tuning vibrational spectroscopy. The native species (DDP-Cl) and the aquated species (DPP-OH) were both observed to bind to the carboxylate group of the serine as evidenced by a frequency shift of 1622-1620 cm-1. However, only DDP-OH was observed to bind to the phosphate group (PO(-)2). The binding of either drug to DMPS resulted in an increased pressure required to halt the reorientational fluctuations of the acyl chains, indicating that the distance between the chains were increased. The two drugs did not partition into the matrix of the hydrophobic section in the model membrane. Collectively, these data suggest that DDP-Cl and DDP-OH are capable of binding to the polar head group of DMPS, resulting in an enlargement of the area of the head and a subsequent increase in the intermolecular distance between the acyl chains.


British Journal of Cancer (1995) 72, 1400–1405
© 1995 Stockton Press All rights reserved 0007–0920/95 $12.00

### Pressure tuning infrared spectroscopic study of cisplatin-induced structural changes in a phosphatidylserine model membrane*

KD Taylor[1], R Goel[1], FH Shirazi[1], M Molepo[1], P Popovic[1], DJ Stewart[1] and PTT Wong[2]

[1]*Ottawa Regional Cancer Centre, Ottawa, Canada K1Y 4K7;* [2]*Department of Biochemistry, University of Ottawa, Ottawa, Canada.*

**Summary** The dynamic effect of *cis*-diamminedichloroplatinum(II) (DPP) and its aquated metabolite (DDP-OH) on a dimyristoylphosphatidylserine (DMPS) model membrane was investigated by pressure tuning vibrational spectroscopy. The native species (DDP-Cl) and the aquated species (DPP-OH) were both observed to bind to the carboxylate group of the serine as evidenced by a frequency shift of 1622–1620 $cm^{-1}$. However, only DDP-OH was observed to bind to the phosphate group ($PO_2^-$). The binding of either drug to DMPS resulted in an increased pressure required to halt the reorientational fluctuations of the acyl chains, indicating that the distance between the chains was increased. The two drugs did not partition into the matrix of the hydrophobic section in the model membrane. Collectively, these data suggest that DDP-Cl and DDP-OH are capable of binding to the polar head group of DMPS, resulting in an enlargement of the area of the head and a subsequent increase in the intermolecular distance between the acyl chains.

**Keywords:** cisplatin; aquated cisplatin metabolite; phosphatidylserine; cell membrane; infrared spectroscopy

The antineoplastic agent, *cis*-diamminedichloroplatinum(II) (DDP) is one of the most actively used chemotherapeutic drugs for the treatment of cancer. However, DDP causes both nephrotoxicity and a peripheral neuropathy. The nephrotoxicity can be alleviated by hydration and the administration of mannitol, but peripheral neuropathy is now often dose limiting (Cersosimo, 1989; Daugaard and Abildgaard, 1989). The neuropathy can be crippling and appears to be related to the cumulative dose of DDP (Borch and Markman, 1989). The mechanism of DDP-induced neurotoxicity is unknown. DDP has been shown to form different metabolites both *in vivo* and *in vitro* (Daley-Yates and McBrien, 1984; Mason *et al.*, 1986*a*, *b*; DeWaal *et al.*, 1987; Goel and Howell, 1989; Mistry *et al.*, 1989). DDP, also termed native cisplatin, is shown in Figure 1a. DDP is the species of drug that is found in clinical formulations and is believed to be responsible for the antineoplastic behaviour. One of the metabolites, termed the aquated species (Figure 1b) because of the replacement of the chloride ions with hydroxo and/or aqua ligands (Miller and House, 1990), was the most nephrotoxic metabolite in mice. Bismethionine cisplatin, with two methionine ligands (Figure 1c), was observed to be at least as toxic to central neuroblastoma cells as DDP (Goel and Howell, 1989). These data suggest that DDP and its metabolites may be selective for the cell types to which they are toxic.

The mechanism by which DDP and its metabolites enter cells is also unknown. It has been generally supposed that cisplatin enters the cell largely through passive diffusion (Gately, 1993). However, other authors have reported that DDP may be actively transported via an amino acid transport system (Gale *et al.*, 1973) which is energy dependent (Andrews *et al.*, 1988) and cAMP mediated (Andrews and Howell, 1990). Thus, there is evidence that supports both passive diffusion and active transport as possible mechanisms of cellular uptake of DDP. Therefore, there is the possibility of an interaction between DDP (or its metabolites) and the macromolecular constituents of the cell membrane that may result in a cytotoxic response.

Phosphatidylserine (PS) constitutes approximately 11–14% of the total phospholipids found in olfactory nerves and synaptic membranes respectively (Chacko *et al.*, 1972; Hitzmann *et al.*, 1986). Bergelson *et al.* (1988) have reported that quantities of PS are elevated in tumour cells, and Mann *et al.* (1988) have implicated this increase of phospholipids in the resistance of tumour cells to cisplatin chemotherapy. High PS concentrations have also been found in several preparations of excitable membranes (Camejo *et al.*, 1969; Chacko *et al.*, 1976), and data obtained from different tissues suggest that PS is present in greater proportions in excitable tissues (Ritchie and Rogart, 1977) than in non-excitable ones (Lazdunski *et al.*, 1980). The structure and thermotropic properties of PS have been shown to be sensitive to the divalent cations, $Ca^{2+}$ (Hauser *et al.*, 1982; Dluhy *et al.*, 1983; Casal *et al.*, 1987*a*, *b*, *c*) and $Mg^{2+}$ (Papahadjopoulos *et al.*, 1977). These studies have concentrated on the thermotropic behaviour of PS with divalent cations.

**Figure 1** Molecular structures of (**a**) cisplatin, (**b**) aquated cisplatin metabolite and (**c**) bismethionine cisplatin metabolite.

Correspondence: R Goel, Ottawa Regional Cancer Centre, Civic Division, 190 Melrose Avenue, Ottawa, Ontario K1A 4K7, Canada

*This work was presented at the annual American Association for Cancer Research meetings in San Diego, CA, USA, May 19-24 1992 and published as an abstract in the Proceedings of the American Association for Cancer Research.

*Issued as NRCC No. 33280.
Received 5 July 1994; revised 14 June 1995; accepted 4 July 1995

With the addition of high pressure as a variable (pressure-tuning vibrational spectroscopy), information on lipid structure and the dynamic interaction with platinum coordinate species can be obtained using Fourier-transformed infrared spectroscopy (FTIR). The infrared spectral parameters and their pressure dependence (particularly the frequencies, widths, intensities and shapes of the infrared bands) are very sensitive to the structural and dynamic properties of biomolecules, as well as to the functional groups in molecules (Wong, 1984; Wong and Mantsch, 1985). Therefore, pressure-tuning vibrational spectroscopy is a powerful technique in the study of biological systems. It provides not only general but also detailed information on the structural and dynamic properties of biological systems at the molecular and functional group level (Wong, 1987).

In this study, we have used FTIR spectroscopy with a pressure effect to monitor the barotropic behaviour of several infrared features of a fully hydrated PS bilayer containing dimyristoylphosphatidylserine [1,2-dimyristoyl-*sn*-glycero-3-phospho-L-serine (DMPS)]. The interaction of the DMPS model membrane with both the native DDP and aquated metabolite was then examined.

### Materials and methods

### *Materials*

DMPS-$Na^+$ was purchased from Avanti Polar Lipids (Birmingham, AL, USA). Crystalline *cis*-diamminedichloroplatinum(II) and Platinol were a gift from Bristol-Myers-Squibb (Evansville, IN, USA) and Cisplatin Injectable was obtained from Horner Laboratories (Montreal, Canada). All other chemicals were of analytical grade.

### *Sample preparation*

Native cisplatin was prepared either from the clinical formulation of Platinol or by dissolving Cisplatin Injectable in saline or $D_2O$/Tris buffer (when observing the phosphate stretching region) to obtain a 1 mg $ml^{-1}$ concentration. An aliquot of 1 mg of DMPS was dispersed in 1% weight of $D_2O$/Tris buffer (which contained no chloride ions) or double-distilled water. Aquated cisplatin was prepared by dissolving crystalline DDP at a 1 mg $ml^{-1}$ concentration in either $D_2O$ (when observing the phosphate stretching region) or double-distilled water, and then heating the solution for 1 h at 37–40°C. The actual amount of DDP added to 1 mg of DMPS is 0.23 mg. The final concentration of DDP will therefore be equal to 230 $\mu$g $ml^{-1}$ (mol. wt. of DDP = 300 g), since the final volume of DDP and DMPS solution is 1 ml. This amount is equal to 1.5 $\mu$M DMPS (mol. wt. of DMPS = 651.82 g) and 0.76 mM DDP (molar ratio of 1:2 for DDP/DMPS). The same molar ratio of DDP-OH/DMPS will result since DDP-OH was prepared from DDP at the above-mentioned concentration in low-chloride media. All the DDP should be converted to a mixture of hydroxo and aqua species after 2 h in a low-chloride environment (Miller and House, 1990). Concentrations in this range were chosen to meet the limits of infrared spectroscopy. This solution was then heated for 1 h at 37–40°C. All DMPS/DDP or DMPS/DDP-OH preparations were heated to 37–40°C for 2 h, vigorously mixed and centrifuged at 12 000 *g* for 10 min. The supernatant was aspirated and the resulting pellet was then stored at 4°C for 72 h. Before FTIR spectroscopy, the DDP-OH/DMPS and DDP-Cl/DMPS/$H_2O$ mixtures were heated to 37°C for 30 min and vigorously mixed. Small amounts of the homogeneous dispersions (typically 0.01 mg) were placed together with powdered α-quartz, in a 0.37 mm-diameter hole in a 0.23 mm-thick stainless steel gasket mounted on a diamond anvil cell, as described previously (Wong *et al.*, 1985).

### *FTIR spectroscopy*

Infrared spectra were measured at 28°C on a Bomem Model Michelson 110 Fourier-transformed spectrophotometer with a liquid nitrogen-cooled mercury cadmium telluride detector. The infrared beam was condensed by a sodium chloride lens onto the diamond anvil cell. For each spectrum, 250 scans were co-added, at a spectral resolution of 4 $cm^{-1}$ (corresponding to a total measuring time of approximately 8 min). Pressures were determined from the 695 $cm^{-1}$ photon band of α-quartz (Wong *et al.*, 1985). The frequency of this band was obtained from third-order derivative spectra using a breakpoint of 0.3 in the Fourier domain and pressures were calculated according to the related equation. In order to separate unresolvable infrared band contours, Fourier derivation techniques (Moffatt *et al.*, 1986) were applied. Frequencies associated with the methylene scissoring and rocking modes were obtained from third-order derivative spectra using a breakpoint of 0.95 in the Fourier domain, while those associated with the carbonyl, carboxylate and phosphate stretching modes were also obtained from third-order derivative spectra but using a breakpoint of 0.3, 0.14 and 0.1 respectively in the Fourier domain.

### Results

Infrared spectra of DMPS bilayers, DDP-OH/DMPS and DDP-Cl/DMPS mixtures have been measured as a function of pressure. The application of pressure is used as a tool to enhance the intermolecular interactions so that additional structural information of a lipid–drug interaction can be obtained. There were four regions of interest in the infrared spectra arising from the work done using these DMPS and cisplatin systems. The regions were the following: (1) 1227 $cm^{-1}$ (antisymmetrical stretching of phosphate); (2) 1621 $cm^{-1}$ (antisymmetrical stretching of the serine carboxylate); (3) 2849 $cm^{-1}$ (symmetrical stretching of methylene); and (4) 1467 $cm^{-1}$ (bending mode of $CH_2$ chain).

### *FTIR of DMPS*

The infrared spectra of the DMPS in the region from 1550 to 1800 $cm^{-1}$ show that there are two bands at approximately 1622 and 1737 $cm^{-1}$ (Auger *et al.*, 1990). The band at 1622 $cm^{-1}$ corresponds to the antisymmetric stretching vibration of the serine carboxylate group (Casal *et al.*, 1987*a*, *b*, *c*) whereas the symmetric stretching vibrations of the serine carboxylate occur between 1390 and 1420 $cm^{-1}$. The band at 1737 $cm^{-1}$ corresponds to the ester carbonyl (C=O) stretching mode of the lipid acyl chains, which have been shown to give characteristic bands in the spectral region of 1725-1745 $cm^{-1}$ (Auger *et al.*, 1990). The ester C=O stretching band did not show any significant variation in shape and intensity as a function of pressure. The band corresponding to serine C=O stretching also did not show significant variation in shape and intensity as a function of pressure up to 16 kbar. Auger *et al.* (1990) have shown that the serine C=O

**Figure 2** Pressure dependence of the frequency of the $\delta CH_2$ mode for DMPS (●), DMPS + DDP-Cl(+) and DMPS + DDP-OH(O) which have been hydrated in $D_2O$.

stretching band does not change up to pressures of approximately 25 kbar, suggesting that the structure of the carboxylate group of DMPS in the gel state is not significantly altered by the application of hydrostatic pressure.

In the region of 1467 cm $^{-1}$, the bending mode of $CH_2$ is observed. The barotropic behaviour of the $CH_2$ group results in a correlation field splitting at a pressure above 3.7 kbars (Figure 2). The pressure-induced correlation field splitting is a result of the interchain interactions between the lipid hydrocarbon chains (Snyder *et al.*, 1961). The presence of a single $CH_2$ band at atmospheric pressure reflects that under those conditions of pressure and temperature, the orientation of the methylene chains is highly disordered owing to significant reorientational fluctuations and torsion/twisting motions of the acyl chains. Increasing the pressure leads to a dampening of these reorientational fluctuations and chain motions and an increase in interchain interactions which gives rise to the observed correlation field splitting. The same correlation field splitting can be seen in the DMPS samples that were hydrated using $D_2O$ and $H_2O$. The pressure at which the splitting occurs is only slightly higher when $H_2O$ was used as the hydrating reagent.

At a frequency of approximately 1227 cm$^{-1}$ the antisymmetric stretching of the phosphate group ($PO_2^-$) is observed with DMPS bilayers. A frequency of 1221 cm$^{-1}$ is associated with a fully hydrated phosphate group, while a band at 1240–1262 cm$^{-1}$ is observed when the group is dehydrated (Wong and Mantsch, 1988). Therefore, the phosphate group of DMPS is almost fully hydrated.

The pH of the samples was approximately 7.0, which was important in determining the ionisation states of the phosphate, serine carboxylate and serine amino groups. Katzman *et al.* (1966) have established that the p$K_a$ of the serine carboxylate and amino groups is approximately 3.0 and 8.0 respectively. Therefore, at pH 7.0 both of these groups are ionised. The presence of a strong band at 1090 cm$^{-1}$ which corresponds to the stretching of a P-O- group also indicates that the phosphate group is ionised (Katzmann *et al.*, 1966).

The pH of the solutions was also important in determining the rate of formation of aquated cisplatin. According to Miller and House (1990), at pH 7.4 with a low chloride ion concentration (4 mM) there will be complete hydrolysis of DDP ($t_{1/2}$ ~2 h at 37°C) to give a 50:50 equilibrium mixture of (chloro)(hydroxo) and (chloro)(aqua) containing compounds. Therefore, in a system devoid of exogenous chloride ions, there will be a complete loss of chloride ligands and an equilibrium mixture of di(hydroxo) and (aqua)(hydroxo) containing compounds will result.

### *The addition of cisplatin to DMPS*

When DDP-OH and DDP-Cl were added to the DMPS bilayers, there was a frequency shift of the antisymmetric stretching of the serine carboxylate (Figure 3) at atmospheric pressure. As the pressure was increased, the frequencies of the serine carboxylate groups from DMPS, DMPS + DDP-OH and DMPS + DDP-Cl changed in a parallel fashion (Figure 4). The frequencies of this band in both DDP-Cl- and DDP-OH treated-DMPS are about the same in the pressure range up to 16 kbars. They are more or less independent of pressure below approximately 8 kbars and

**Figure 3** Plot of infrared spectra of DMPS, DMPS + DDP-Cl and DMPS + DDP-OH in the region of the antisymmetric $CO_2^-$ stretching band at atmospheric pressure.

**Figure 4** Pressure dependence of the frequency of the $\nu_{as}$ ($CO_2^-$) for DMPS (●), DMPS + DDP-CL (+) and DMPS + DDP-OH (O).

**Figure 5** Diagrammatic representation of the hypothetical binding model of DDP-Cl to the carboxylate group of serine. The same model would represent the binding of DDP-OH to the carboxylate group.

**Figure 6** Pressure dependence of the frequency of the $\nu_s$ ($CH_2$) mode for DMPS (●), DMPS + DDP-Cl (+) and DMPS + DDP-OH (O).

increase slightly with increasing pressure above 8 kbars. The frequency of the symmetrical stretching of the C=O bond from the serine carboxylate group was observed to shift from 1320 to 1310 $cm^{-1}$ following the addition of DDP (data not shown).

It was observed that the frequency of the band corresponding to the carboxylate group decreased in a linear fashion as a function of pressure. After 3 days of DDP incubation, the changes in frequency seen with DDP-Cl and DDP-OH were the same. A 10 day incubation of DMPS with either DDP-OH or DDP-Cl resulted in a maximum shift in band frequency to 1615 $cm^{-1}$. After 5–6 days there was a transition state observed in both samples with two peaks appearing at 1618 and 1615 $cm^{-1}$.

The pressure at which the correlation field splitting occurs is significantly increased when DMPS is treated with DDP-OH or DDP-Cl. When DDP-OH or DDP-Cl are added to DMPS hydrated with $D_2O$, the pressure required to induce correlation field splitting is increased by 1.1 and 1.0 kbars respectively (Figure 2). The addition of either drug to DMPS hydrated with water results in similar correlation field splitting pressures.

The frequencies of the symmetrical ($\alpha_s$) and antisymmetrical ($\alpha_{as}$) stretching modes of the methyl ($CH_2$) and methylene ($CH_3$) groups were monitored for changes. In particular, the $\alpha_s$ ($CH_2$) at 2849 $cm^{-1}$ was monitored for any frequency *vs* pressure variation. Figure 6 shows the relationship between DMPS and DMPS with either DDP-OH or DDP-Cl. With either form of cisplatin, there was no significant variation in the $\alpha_s$ ($CH_2$) within the experimental errors when compared with DMPS alone.

As shown in Figure 7, at atmospheric pressure the band corresponding to the antisymmetric stretching of $PO_2^-$ becomes very broad and the centre of gravity of that band is slightly decreased when DDP-OH is present. The band is also asymmetrical indicating that there is another band overlapping on the low frequency side of the main band. The spectra of the DMPS and DMPS + DDP-Cl show that there is only one main band. The main band in all three systems corresponds to $PO_2^-$ hydrogen bonded to water. The overlapping band in the DMPS + DDP-OH system corresponds to the stronger hydrogen bonds between $PO_2^-$ and the OH groups of DDP-OH that form by displacing the existing hydrogen-bound water.

### Discussion

The gel (Lβ') to liquid-crystalline (L) phase transition temperature for DMPS is 39°C (Casal *et al.*, 1987*b*). At ambient temperature (28°C) and pressure DMPS is in the gel phase, indicating that all of the pressure-induced effects observed in our studies were in the DMPS gel phase.

**Figure 7** Plot of the infrared spectra of DMPS, DMPS + DDP-Cl and DMPS + DDP-OH in the region of the antisymmetrical stretching mode of $PO_2^-$ at atmospheric pressure.

The infrared spectra of both aquated and native cisplatin were obtained at atmospheric pressure. Both spectra showed that the infrared bands of cisplatin did not overlap with the bands associated with DMPS. Therefore, the presence of the cisplatin does not interfere with the IR spectra containing DMPS and either metabolite.

A lower frequency observed in the DDP-treated DMPS samples indicates that both DDP-OH and DDP-Cl are bound to the carboxylate group of serine. This frequency change of the peak suggested that the DDP/DMPS interaction may involve the binding of the platinum(II) to the C-O- portion of the carboxylate group. Figure 5 diagrammatically represents the potential binding kinetics. The binding of platinum to the C-O- results in a flow of electrons from the C=O of the carboxylate group towards the Pt-O-C group. This causes a decrease in the electron density of the -C=O bond and results in a weaker -C=O bond and hence a decrease in -C=O stretching frequency of the serine carboxylate group. The binding of DDP to the serine carboxylated group is a time-dependent effect. Data suggest that the binding of native or aquated cisplatin to the carboxylate group is sterically hindered initially by other functional groups in the polar head. However, over a period of 10 days there is a yielding of these steric hindrances that leads to a decrease in the distance between cisplatin and the carboxylate group. The close proximity of cisplatin and the carboxylate group results in a stronger bond that is reflected in a further decrease in frequency. Therefore, the binding of cisplatin to DMPS involves a kinetic process. The interaction of DMPS with DDP seems to be very slow and difficult (even after about 2 h exposure), however, cellular uptake of DDP is very fast and a significant amount of DDP may accumulate in the cell within a few minutes (Gately and Howell, 1993). Therefore, we suggest that the priority in DDP uptake which is quite rapid *in vivo*, is not through its interaction with DMPS.

An indication of the orientational disorder of the acyl chains in hydrated DMPS can be obtained from the pressure dependencies of the $CH_2$ bending band. Correlational field splitting arises when high pressure immobolises the two hydrocarbon chains approximately perpendicularly to each other (Wong and Mantsch, 1988). Results indicate that binding of DDP to DMPS bilayers decreases the orientational ordering of the DMPS acyl chains, and thus increases the pressure necessary to stop the acyl chain reorientational fluctuations and torsion/twisting motions and to induce a correlation field splitting.

DDP-OH and DDP-Cl do not directly interact with the acyl chains in the hydrophobic portion of the lipid bilayer, since there was no significant variation in the $\alpha_s$ ($CH_2$) within the experimental errors when compared with DMPS alone. Instead, cisplatin acts indirectly to modify the reorientational fluctuations of the acyl chains.

The addition of DDP-OH but not DDP-Cl to DMPS affects the stretching mode of the phosphate group ($PO_2^-$). Stronger DDP-OH/$PO_2^-$ binding results in a decrease in the frequency of the $PO_2^-$ stretching mode. Therefore, in the DMPS + DDP-OH system, $PO_2^-$ will be partially hydrogen-bonded to both water and DDP-OH.

A hypothetical model of the cisplatin/DMPS interaction has been developed to explain the results obtained from these experiments. According to the arrangement of the polar head group, the carboxylate group is the first to come into contact with cisplatin. Both the native and aquated species attach to the carboxylate group through the platinum (II) atom. Owing to its increased polarity (hydrophilicity), the aquated species binds tightly to the head group by interacting with the phosphate group. Conversely, DDP-Cl, which is neutral, does not interact with the phosphate group, and binding to the head group is not as great as the aquated species. The binding of cisplatin to the carboxylate and/or phosphate groups has the effect of enlarging the polar head, which causes an increase in the intermolecular distances of the acyl chains. The increased distance between acyl chains results in greater disorder and hence greater reorientational fluctuations and torsion/twisting motions. Therefore, the pressure required to halt the increased chain motions is increased. The pressures required to stop acyl chain motions were consistently higher with the DDP-OH-treated bilayers. It is possible that as DDP-OH diffuses into the head and binds to the phosphate group, the size of the polar head reaches a maximum size which is reflected in an increased correlation field splitting pressure.

When cisplatin is administered as an antineoplastic agent, cisplatin is maintained in the native conformation by preparing the drug with 0.9% sodium chloride. The conversion of native DDP to aquated cisplatin is proposed to occur in the cytoplasm of cells, presumably because of the low concentration of chloride ions (LeRoy, 1975). In fact the extracellular and intracellular concentrations of chloride ion across a neuronal somal membrane are approximately 107 and 8 mEq $l^{-1}$ respectively. The data reported in this paper would suggest that neither of the cisplatin metabolites has the ability to passively diffuse through a phosphatidylserine lipid bilayer. Instead, the metabolites interact with the polar head groups and increase the distance betwen acyl chains. We have also shown that cisplatin could diffuse through a relatively fluid model membrane made from phosphatidylcholine (PC), but could not diffuse into more rigid model membranes made from phosphatidylethanolmine (PE) (Taylor *et al.*, 1993; Stewart *et al.*, 1994). In addition to PS, both PE and PC are of interest because concentrations of PE and PS are reported to be increased in the plasma membrane of DDP-resistant tumour cells, and concentrations of PC are reported to be very high in the plasma membranes of DDP-sensitive cells (Mann *et al.*, 1988).

On the basis of the data presented in this paper and the physical properties of native and aquated cisplatin, we propose that it would be difficult for native DDP or aquated cisplatin to passively diffuse through a plasma membrane (PM) that contained a large quantity of phosphatidylserine. We hypothesise that one factor that may be involved in the relatively low intracellular cisplatin uptake in some DDP resistant cell lines and tumours is their increased content of PS. Furthermore, for effective intracellular accumulation of cisplatin, the PM would have to contain a phospholipid such as phosphatidylcholine that would allow DDP to pass through and/or a transport mechanism such as an integral protein. Once inside the low chloride environment of the cell, replacement of the chloro ligands with other ligands may occur, which may result in cytotoxic response to the cell.

Although it is proposed that the mechanism of DDP-induced cytotoxicity against cancer cells is through its binding to DNA (Andrews and Howell, 1990), the mechanisms of DDP-induced nephrotoxicity and neurotoxicity are not known (Daugaard and Abildgaard, 1989; Cersosimo, 1989). There is some evidence to suggest that at least some of the DDP-induced damage in the kidney occurs in the cell membranes, with renal ATPase activity being affected (Daley-Yates and McBrien, 1982; Uozomi and Litterest, 1985). *In vivo*, there is evidence that the aquated cisplatin metabolites are responsible for a significant part of nephrotoxicity caused by clinical DDP administration (Mistry *et al.*, 1989; Jones *et al.*, 1991). We have shown that aquated DDP binds more avidly than native DDP to the DMPS bilayer. It may be that the mechanism of DDP-induced nephrotoxicity in humans is related to the disruption of the cell membrane by aquated DDP through their interaction with phospholipids such as DMPS.

**Acknowledgements**
The authors gratefully acknowledge the technical assistance from D Moffatt, Bristol-Myers-Squibb for the gifts of crystalline cisplatin and Platinol and to the National Cancer Institute of Canada for funding. This research was supported by the National Cancer Institute of Canada with funds from the Canadian Cancer Society, and in part by the Ministry of Health, Treatment and Medical Education, Government of the Islamic Republic of Iran.

### References

ANDREWS PA AND HOWELL SB. (1990). Cellular pharmacology of cisplatin: perspectives on mechanisms of acquired resistance. *Cancer Cells*, **2**, 35–43.

ANDREWS PA, VELURY S, MANN SC AND HOWELL SB. (1988). *Cis*-diamminedichloroplatinum(II) accumulation in sensitive and resistance human ovarian carcinoma cells. *Cancer Res.*, **48**, 68–73.

AUGER M, SMITH ICP, MANTSCH HH AND WONG PTT. (1990). High pressure infrared study of phosphatidylserine bilayers and their interactions with the local anesthetic tetracaine. *Biochemistry*, **29**, 2008–2015.

BERGELSON LD, DYATLOVITSKAYA EV, TORKHOVSKAYA TI, SOROKINA LB AND GORKOVA NP. (1988). Phospholipid composition of membranes in the tumor cell. *Biochim. Biophys. Acta*, **210**, 287–298.

BORCH RF AND MARKMAN M. (1989). Biochemical modulation of cisplatin toxicity. *Pharmacol. Ther.*, **41**, 371–380.

BYFIELD JE AND CALABRO-JONES PM. (1982). Further evidence for carrier-mediated cell uptake of cis-dichlorodiamine platinum (CDDP). (abstract). *Proc. Am. Assoc. Cancer Res.*, **23**, 167.

CAMEJO G, VILLEGAS GM, BARNOLA FV AND VILLEGAS R. (1969). Characterization of two different membrane fractions isolated from the first stellar nerves of the squid *Dosidicus gigas*. *Biochim. Biophys. Acta*, **193**, 247–259.

CASAL HL, MANTSCH HH, PALTAUF F AND HAUSER H. (1987*a*). Infrared studies of fully hydrated saturated phosphatidylserine bilayers. Effects of $Li^+$ and $Ca^+$. *Biochim. Biophys. Acta*, **919**, 275–286.

CASAL HL, MANTSCH HH AND HAUSER H. (1987*b*). Infrared and 31P-NMR studies of the interaction of $Mg^{2+}$ with phosphatidylserines: effect of hydrocarbon chain unsaturation. *Biochemistry*, **26**, 4408–4416.

CASAL HL, MARTIN A, MANTSCH HH, PAULTAUF F AND HAUSER H. (1987*c*). Infrared studies of fully hydrated unsaturated phosphatidylserine bilayers. Effects of $Li^+$ and $Ca^+$. *Biochemistry*, **26**, 7395–7401.

CERSOSIMO RJ. (1989). Cisplatin neurotoxicity. *Cancer Treat. Rev.*, **16**, 195–211.

CHACKO GK, GOLDMAN DE AND PENNOCK BE. (1972). Composition and characterization of the lipids of garfish (*Lepisosteus osseus*) olfactory nerve, a tissue rich in axonal membrane. *Biochim. Biophys. Acta*, **280**, 1–16.

CHACKO GK, VILLEGAS GM, BARNOLA FV, VILLEGAS R AND GOLDMAN DE. (1976). The polypeptide and the phospholipid components of axon plasma membranes. *Biochim. Biophys. Acta*, **443**, 19–32.

DALEY-YATES PT AND MCBRIEN DCH. (1982). The inhibition of real ATPase by cisplatin and some biotransformation products. *Chem. Biol. Interact.*, **40**, 325–334.

DALEY-YATES PT AND MCBRIEN DCH. (1984). Cisplatin metabolites in plasma, a study of their pharmacokinetics and importance in the nephrotoxic and antitumour activity of cisplatin. *Biochem. Pharmacol.*, **33**, 3063–3080.

DAUGAARD G AND ABILDGAARD U. (1989). Cisplatin nephrotoxicity. *Cancer Chemother. Pharmacol.*, **25**, 1–9.

DEWAAL WAJ, MAESSEN FJMJ AND KRAAK JC. (1987). Analysis of platinum species originating from cis-diamminedichloroplatinum-(II) (cisplatin) in human and rat plasma by high-performance liquid chromatography with on-line inductively coupled plasma atomic emission spectrometric detection. *J. Chromatogr.*, **407**, 253–272.

DLUHY RA, CAMERON DG, MANTSCH HH AND MENDELSON R. (1983). Interaction of dipalmitoylphosphatidylcholine and dimyristoylphosphatidylcholine-d54 mixtures with glycophorin. A fourier transform infrared investigation. *Biochemistry*, **22**, 6318-6325.

GALE GR, MORRIS CR, ATKINS LM AND SMITH AB. (1973). Binding of an antitumor platinum compound to cells as influenced by physical factors and pharmacologically active agents. *Cancer Res.*, **33**, 813–818.

GATELY DP AND HOWELL SB. (1993). Cellular accumulation of the anticancer agent cisplatin: A review. *Br. J. Cancer*, 1170–1176.

GOEL R AND HOWELL SB. (1989). Differential effect of cisplatin metabolites on various cell types. (abstract). *Proc. Am. Assoc. Cancer Res.*, **30**, 466.

HAUSER H, PALTAUF F AND SHIPLEY GG. (1982). Structure and thermotropic behaviour of phosphatidylserine bilayer membranes. *Biochemistry*, **21**, 1061–1067.

HITZMANN RJ, SCHUELER HE, GRAHAM-BRITTAIN C AND KREISHMAN GP.(1986). Ethanol-induced changes in neuronal membrane order. *Biochim. Biophys. Acta.*, **859**, 189–197.

JONES MM, BASINGER MA, BEATY JA AND HOLSCHER MA. (1991). The relative nephrotoxicity of cisplatin. cis-[Pt(NH3)2(guanosine)2]2+, and the hydrolysis product of cisplatin in rat. *Cancer Chemother. Pharmacol.*, **29**, 29–32.

KATZMANN R, DI LUZIO FC, GILLIAM WS AND KOTCH A. (1966). *Research and Development Progress Report*. No. 178, pp. 1–77. United States: Department of the Interior.

LAZDUNSKI M, BALERNA M, BARHANIN J, CHICHEPORTICHE R, FOSSETT M, FRELIN C, JACQUES Y, LOMBERT A, POYSSEGUR J, RENAUD JF, ROMERY G, SCHWEITZ H AND VINCENT JP. (1980). Molecular aspects of the structure and mechanism of the voltage-dependent sodium channel. *Ann. N.Y. Acad. Sci.*, **358**, 169–182.

LEROY AF. (1975). Interactions of platinum metals and their complexes in biological systems. *Environ. Health Perspect.*, **10**, 73–83.

MANN SC, ANDREWS PA AND HOWELL SB. (1988). Comparison of lipid content; Surface membrane fluidity, and temperature dependence of cis-diamminedichloroplatinum II accumulation in sensitive and resistant human ovarian carcinoma cells. *Anti Cancer Research*, **8**, 1211–1216.

MASON RW, HOGG S AND EDWARDS IR. (1986*a*). Distribution of Pt in the urine and kidney of the cisplatin treated rat. *Toxicology*, **38**, 219–221.

MASON RW, HOGG S AND EDWARDS IR. (1986*b*). Time course of the binding of platinum to subfractions of the kidney cytosol in the cisplatin-treated rat. *Res. Commun. Chem. Pathol. Pharmacol.*, **52**, 51–58.

MILLER SE AND HOUSE DA. (1990). The hydrolysis products of *cis*-diamminedichloroplatinum(II); 6: a kinetic comparison of the *cis*- and *trans*-isomers and other *cis*-di(amine)di(chloro)platinum-(II) compounds. *Inorganica Chim. Acta*, **173**, 53–60.

MISTRY P, LEE C AND MCBRIEN DCH. (1989). Interacellular metabolites of cisplatin in the rat kidney. *Cancer Chemother. Pharmacol.*, **24**, 73–79.

MOFFATT DJ, KAUPPINEN JK, CAMERON DG, MANTSCH HH AND JONES RN. (1986). Computer programs for infrared spectrophotometry. *NRCC Bull.* No. 18.

PAPAHADJOPOULOS D, VAIL WJ, NEWTON C, NIR S, JACOBSON K, POSTE G AND LAZO R. (1977). Studies on membrane fusion. III. The role of calcium-induced phase changes. *Biochim. Biophys. Acta*, **465**, 579–598.

RITCHIE JM AND ROGART RB. (1977). The binding of saxitoxin and tetrodotoxin to excitable tissue. *Rev. Physiol. Biochem. Pharmacol.*, **79**, 1–50.

SNYDER RG. (1961). Vibrational spectra of crystalline n-paraffins. II. Intramolecular affects. *J. Mol. Spectrosc.*, **7**, 116–144.

STEWART DJ, MOLEPO M, EAPEN L, MONTPETIT V, GOEL R, WONG P, POPOVIC P, TAYLOR K AND RAAPORST GP. (1994). Cisplatin and radiation in the treatment of tumors of the central nervous system: Pharmacological considerations and results of early studies. *Int. J. Radiation Biol. Phys.*, **28**, 531–542.

TAYLOR KD, GOEL R, STEWART DJ AND WONG PTT. (1993). Pressure tuning spectroscopic of cisplatin-induced changes in a phosphatidylcholine model membrane. (abstract). *Proc. Am. Assoc. Cancer Res.*, **34**, 2389.

UOZOMI J AND LITTEREST CL. (1985). The effect of cisplatin on renal ATPase activity in vivo and in vitro. *Cancer Chemother. Pharmacol.*, **15**, 93–96.

WONG PTT. (1984). Raman spectroscopy of thermotropic and high-pressure phases of aqueous phospholipid dispersions. *Ann. Rev. Biophys. Bioeng.*, **13**, 1–24.

WONG PTT AND MANTSCH HH. (1985). Pressure effects on the infrared spectrum of 1,2-dipalmitolycholine bilayers in water. *J. Chem. Phys.*, **83**, 3268–3274.

WONG PTT. (1987). Vibrational spectroscopy under high pressure. *Curr. Perspect. High Press. Biol.*, **22**, 287–314.

WONG PTT AND MANTSCH HH. (1988). High-pressure infrared spectroscopic evidence of water binding sites in 1,2-diacyl phospholipids. *Chem. Phys. Lipids*, **46**, 213–224.

WONG PTT, MOFFATT, DJ AND BAUDAIS FL. (1985). Crystalline quartz as internal pressure calibrant for high-pressure spectroscopy. *Appl. Spectrosc.*, **39**, 733–735.